# Supplementary material for: The causal role of circulating inflammatory markers in osteoporosis: a bidirectional Mendelian randomized study
Source: Front Immunol. 2024 Jul 18;15:1412298. doi: 10.3389/fimmu.2024.1412298 (PMC11291241; doi:10.3389/fimmu.2024.1412298)
Supplement: Supplementary file 1 [file DataSheet_1.zip › supplementary material/CIM-OSTEOPOROSIS /GCST90274784.csv_leave_one_out.pdf]

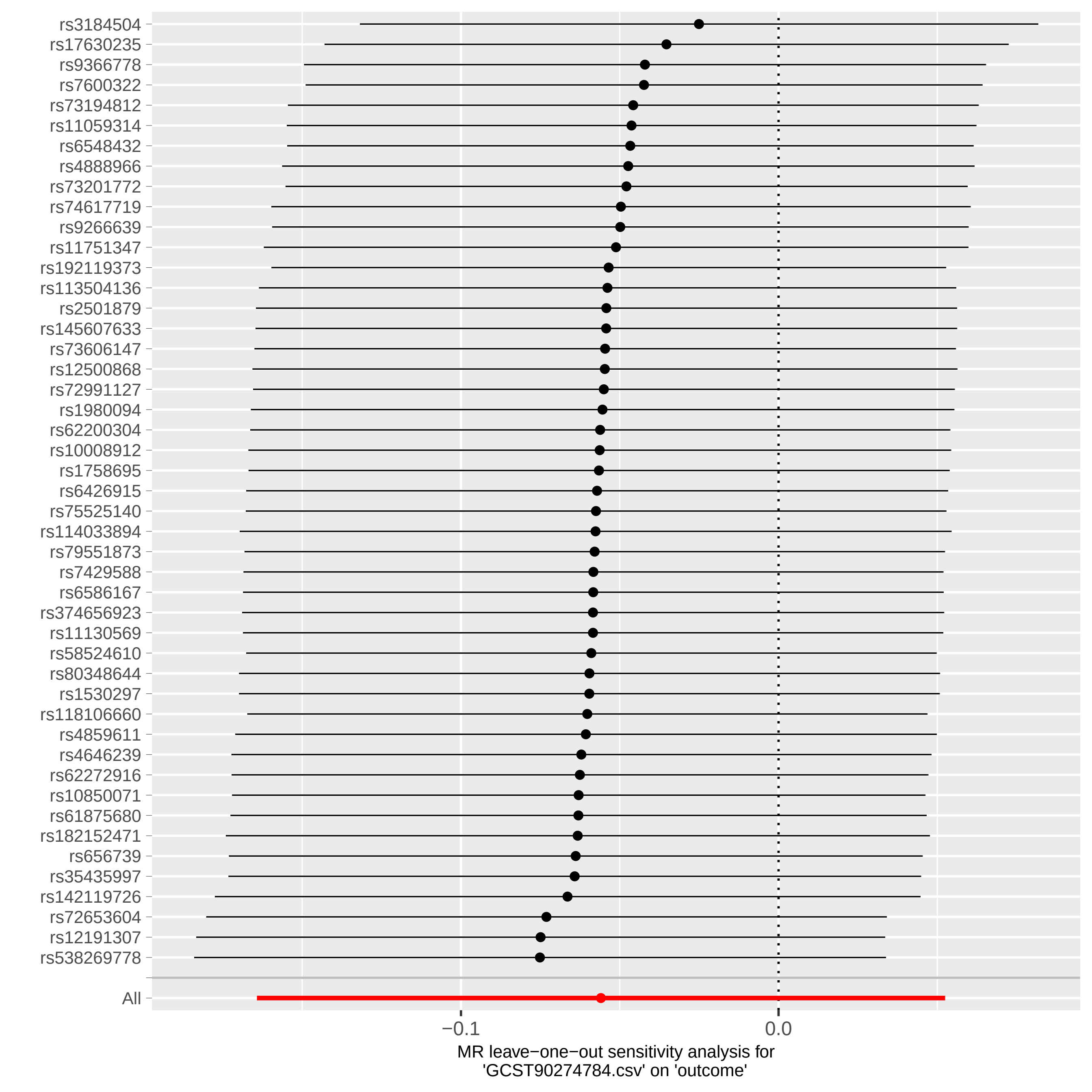
rs3184504
rs17630235
rs9366778
rs7600322
rs73194812
rs11059314
rs6548432
rs4888966
rs73201772
rs74617719
rs9266639
rs11751347
rs192119373
rs113504136
rs2501879
rs145607633
rs73606147
rs12500868
rs72991127
rs1980094
rs62200304
rs10008912
rs1758695
rs6426915
rs75525140
rs114033894
rs79551873
rs7429588
rs6586167
rs374656923
rs11130569
rs58524610
rs80348644
rs1530297
rs118106660
rs4859611
rs4646239
rs62272916
rs10850071
rs61875680
rs182152471
rs656739
rs35435997
rs142119726
rs72653604
rs12191307
rs538269778
All
−0.1
0.0
MR leave−one−out sensitivity analysis for
'GCST90274784.csv' on 'outcome'
